# Neuroglial-Breast Cancer Crosstalk Shapes the Brain Metastatic Niche

**DOI:** 10.3390/cells15080735

**Published:** 2026-04-21

**Authors:** Sabine Hombach-Klonisch, Eric Hall, Reem Amin, Emily Fedora, Jerry Vriend, Marshall Pitz, Thomas Klonisch

**Affiliations:** 1 Departments of Human Anatomy and Cell Science, Max Rady College of Medicine, Rady Faculty of Health Sciences, University of Manitoba, Winnipeg, MB R3E 0J9, Canada; sabine.hombach-klonisch@umanitoba.ca (S.H.-K.); eric.hall@umanitoba.ca (E.H.); ramin361@mtroyal.ca (R.A.); fedorae@myumanitoba.ca (E.F.);; 2 Departments of Pathology, Max Rady College of Medicine, Rady Faculty of Health Sciences, University of Manitoba, Winnipeg, MB R3E 0J9, Canada; 3 Departments of Medical Microbiology and Infectious Diseases, Max Rady College of Medicine, Rady Faculty of Health Sciences, University of Manitoba, Winnipeg, MB R3E 0J9, Canada; marshall.pitz@umanitoba.ca; 4 CancerCare MB, Winnipeg, MB R3E 0V9, Canada; 5 Departments of Internal Medicine, Max Rady College of Medicine, Rady Faculty of Health Sciences, University of Manitoba, Winnipeg, MB R3E 0J9, Canada

**Keywords:** breast cancer brain metastasis (BCBM), triple negative breast cancer (TNBC), HER2+ breast cancer, neuronal mimicry, tripartite synapse, GABAergic, GABA shunt, glutamatergic, NMDAR, AMPAR, voltage-gated calcium channels (VGCCs), cytonemes, tunneling nanotubes (TNTs), tumor microtubes (TMs)

## Abstract

Breast cancer brain metastasis (BCBM) affects up to 30% of patients with metastatic disease and carries a median survival of only 4–18 months. Emerging evidence reveals that BCBM cells are not passive survivors, but active participants that hijack core neurotransmitter networks, GABA (gamma-aminobutyric acid) and glutamate, to fuel their growth. BCBM, particularly triple-negative breast cancer (TNBC), frequently switch to a GABAergic mode utilizing brain-derived GABA as an oncometabolite. In parallel, BCBM cells can also form direct synapses with neurons, tapping into excitatory input through glutamatergic receptors to drive tumor cell proliferation and survival. Concurrently, reprogrammed astrocytes establish gap junctions, secrete growth factors, and provide metabolic support. Together, tumor cells, neurons, and astrocytes form a pathological partnership locked in feedback loops sustaining metastatic progression. This review focuses on the unique mechanisms employed by distinct breast cancer subtypes and maps the metastatic progression from pre-metastatic to mature brain metastatic niche formation of BCBM. We highlight opportunities to repurpose neurological drugs to disrupt these communication axes.

## 1. Introduction

### 1.1. The Clinical Challenge of Breast Cancer Brain Metastasis

Breast cancer brain metastasis (BCBM) is a devastating complication affecting 10–30% of patients with metastatic disease, with incidence strongly dependent on molecular subtype [1,2,3,4]. The propensity for the colonization of organs outside the breast is highest for Triple-Negative Breast Cancer (TNBC; 25–46%) and HER2+ BC disease (30–55%), while luminal subtypes have lower rates (5–15%) [1,2,3,4]. Nearly one-third of patients with TNBC or HER2+ metastatic breast cancer will eventually develop brain metastases, with an incidence approaching 13% per patient-year [4,5]. Paradoxically, this growing burden of BCBM in the clinic reflects improved systemic control of the extracranial breast cancer disease, which prolongs survival long enough for central nervous system (CNS) involvement to manifest [3,4].

**Figure 1:**
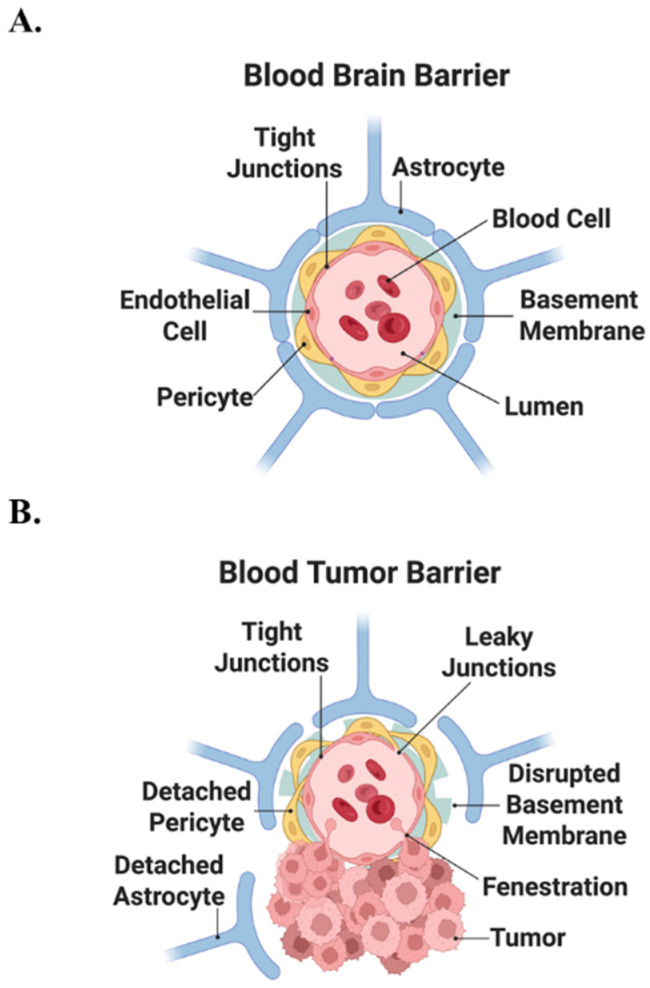
Structural features of the intact blood–brain barrier (BBB) and the blood–tumor barrier (BTB). (**A**) The physiological BBB consists of non-fenestrated brain microvascular endothelial cells sealed by continuous tight junctions, closely associated pericytes embedded in a specialized basement membrane and ensheathing astrocytic end feet. This restricts paracellular diffusion and tightly regulates exchange between the vascular lumen and the neural parenchyma. (**B**) In the BTB, tumor-associated remodeling of this neurovascular unit leads to focal loosening of tight junctions and the formation of leaky junctions and endothelial fenestrations, detachment or loss of pericytes and astrocyte end feet, and disruption of the basement membrane. Collectively, the result is increased but spatially heterogeneous vascular permeability at the tumor-brain interface.

Once BCBM is diagnosed, the prognosis is grim [6,7]. Median overall survival is 3.7–4.9 months for TNBC, 7.1–18.9 months for luminal subtypes, and 13.1–16.5 months for HER2+ BCBM disease [1,2,3,6]. The neurological morbidity of headaches, fatigue, seizures, cognitive impairment, personality changes, and paralysis severely diminishes quality of life [1,2,3,4]. Current treatments such as surgical resection, stereotactic radiosurgery, and whole-brain radiotherapy are largely palliative. Although newer systemic agents cross the blood-brain barrier (BBB) (Figure 1A) or blood-tumor barrier (BTB) (Figure 1B), therapeutic options remain limited [1,6,8]. BTB permeability is highly heterogeneous within and between lesions, causing uneven drug delivery that promotes therapeutic resistance (Figure 1A,B) [8].

### 1.2. Paradigm Shifts in Understanding the Brain Metastatic Niche

Traditionally, the CNS has been regarded as a non-permissive environment for metastasis, protected by an intact BBB and nutrient-restricted milieu. Successful BCBM cells were considered rare survivors capable of bypassing the BBB and persisting in an inhospitable sanctuary.

Contrary to this earlier paradigm, the biology of BC cells in the brain is now recognized as highly dynamic and deeply integrated into neural circuitry. BCBM cells do not simply tolerate the brain environment; they actively engage it by exploiting endogenous neurotransmitter systems [9]. This conceptual shift is supported by the discovery that human BCBM cells upregulate receptors and transporters for the principal inhibitory neurotransmitter GABA (gamma-aminobutyric acid), enabling uptake and catabolism of GABA as a metabolic fuel and oncometabolite via the GABA shunt [9,10,11]. A second paradigm shift arose from evidence that metastatic BC cells, together with neurons and astrocytes, form a reciprocal tripartite signaling network to usurp synaptic mechanisms of neurotransmitter release and uptake [12,13,14,15,16].

Brain metastatic cells frequently reside in peri-synaptic spaces adjacent to glutamatergic neurons or even form direct synaptic contacts [13,15,16]. BCBM cells can express functional NMDA (N-methyl-D-aspartate)–type glutamate receptors on their membranes. This allows BCBM cells to intercept neuronal glutamate and initiate Ca^2+^-dependent signaling cascades that promote their proliferation and survival [16]. Ultrastructural and electrophysiological analyses demonstrate that metastatic cells establish *bona fide* synapses with neurons and receive direct neurotransmitter input [16,17,18]. These synaptically integrated tumor cells express canonical synaptic proteins typically restricted to neurons, including postsynaptic scaffolding molecules and synaptic adhesion proteins [12,13,14,15,18,19]. This neuronal mimicry permits brain metastatic cells to integrate into CNS circuits where direct synaptic input enhances growth, promotes migration, and confers resistance to therapy [15,16,17,18,19].

### 1.3. Scope and Objectives

This review examines adaptive strategies that enable BC cells to colonize and thrive in the brain microenvironment. During the early phase of establishing the brain metastatic niche, GABAergic metabolic adaptation dominates. As the structural and functional integration of metastatic cells into the brain matrix matures, emerging glutamatergic input and increasing synaptic integration foster strong proliferative signaling [9,18]. Where data permit, we emphasize subtype-specific differences between TNBC, HER2+, and luminal BCBM. We consider the temporal evolution of neuron–astrocyte–tumor interactions from pre-metastatic niche conditioning to established lesions and discuss how these insights may guide repurposing of neurological drugs and development of novel therapeutic strategies.

## 2. The Tripartite Synapse: Architecture and Hijacking

### 2.1. Tripartite Synapse Architecture

**Figure 2:**
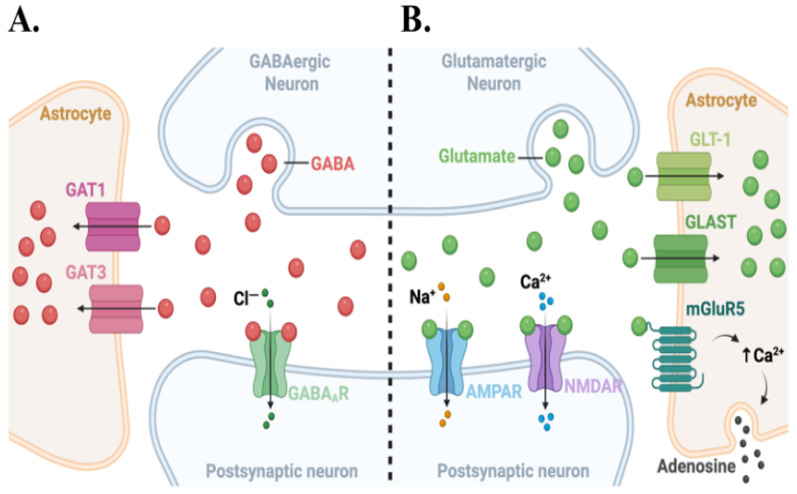
Schematic representation of GABAergic (**A**) and glutamatergic (**B**) tripartite synapses and astrocyte-mediated neurotransmitter clearance. (**A**) At GABAergic synapses, presynaptic release of GABA activates postsynaptic GABA_A_ receptors to promote Cl^−^ influx, while peri-synaptic astrocytes remove GABA via high-affinity GAT1 and GAT3 transporters to terminate inhibitory signaling. (**B**) At glutamatergic synapses, presynaptic glutamate release engages postsynaptic AMPA and NMDA receptors to drive Na^+^ and Ca^2+^ entry, respectively. Astrocytes rapidly take up glutamate spill-over through GLT-1 and GLAST transporters and sense synaptic glutamate via mGluR5, leading to intracellular Ca^2+^ elevations and downstream gliotransmitter (e.g., adenosine) signaling.

The traditional view of a synapse being a contact site for two neurons to communicate has been replaced by the “tripartite synapse” model, which recognizes astrocytes as integral partners (Figure 2) [12,14,20]. In the hippocampus and cortex, astrocytic processes embrace 50–70% of all synapses, positioning astrocytic membranes within nanometers of the synaptic cleft [14,20]. These peri-synaptic processes are studded with receptors and transporters that detect neurotransmitters, allowing astrocytes to listen to and decode neural activity [20,21,22].

During excitatory transmission, astrocytic glutamate transporters GLT-1/EAAT2 and GLAST/EAAT1 rapidly clear glutamate, preventing neurotoxicity and maintaining high signal-to-noise transmission (Figure 2A) [23,24]. Astrocytes similarly regulate extracellular GABA via GAT1/3 transporters to modulate the inhibitory tone (Figure 2A) [25]. Neurotransmitter binding and activation of astrocytic metabotropic receptors, including metabotropic glutamate (Figure 2B), purinergic (P2Y), and GABA_B_ receptors, elevates intracellular Ca^2+^ levels primarily through G-protein-coupled phospholipase-C (PLC) activation and IP_3_-dependent release of Ca^2+^ from internal stores [26]. Astrocytes can propagate these intracellular Ca^2+^ signals through gap-junctional networks to coordinate Ca^2+^-sensitive molecular activities across larger brain territories [27,28]. Elevated astrocytic Ca^2+^ (e.g., via activation of NMDARs) also promotes the release of gliotransmitters, such as glutamate, D-serine, ATP, and adenosine. These astrocyte-derived gliotransmitters modulate synaptic strength and plasticity at nearby neurons (Figure 2B) [13,29]. D-serine acts as a co-agonist at the glycine site of NMDARs, which is essential for full activation. ATP is rapidly degraded to adenosine, which can dampen neurotransmission via presynaptic A1 receptors [30,31]. It is through these mechanisms that astrocytes function as bidirectional tuners that sense neuronal activity and dynamically adjust synaptic efficacy.

### 2.2. Synaptic Plasticity Involves Long-Term Potentiation (LTP) and Depression (LTD)

Synaptic plasticity is a dynamic molecular process that controls activity-dependent changes in synaptic strength and encompasses both long-term potentiation (LTP) and long-term depression (LTD) associated with learning and memory [32,33]. High-frequency stimulation relieves the Mg^2+^ block of NMDARs, causing sustained Ca^2+^ influx to activate Ca^2+^/calmodulin-dependent protein kinase II (CaMKII) [34,35,36]. Activated CaMKII phosphorylates AMPAR subunits, such as GluA1 at Ser831. This enhances channel conductance and promotes AMPAR insertion into the postsynaptic membrane in concert with protein kinase A (PKA) activity [37,38,39]. Conversely, low-frequency stimulation produces smaller, prolonged Ca^2+^ elevations that favor protein phosphatases, such as calcineurin and PP1, which dephosphorylate AMPARs and trigger endocytosis, resulting in reduced synaptic strength [40,41]. Efficient NMDAR activation during LTP requires astrocyte-derived D-serine, while astrocytic glutamate and ATP-derived adenosine assist in further tuning synaptic responses (Figure 2B) [30,42,43]. Tumor cells at the tripartite synapse engage these same receptor systems and molecular mechanisms regulating synaptic plasticity, thereby gaining access to an adaptable signaling framework evolved for learning and memory.

### 2.3. Brain Metastatic Cells Exploit the Synaptic Structure and Function

Upon entering the brain, BCBM cells are not passive bystanders but exploit synaptic signaling using three strategies: peri-synaptic positioning, direct synapse formation, and neuronal mimicry [13,15,16,19].

**Figure 3:**
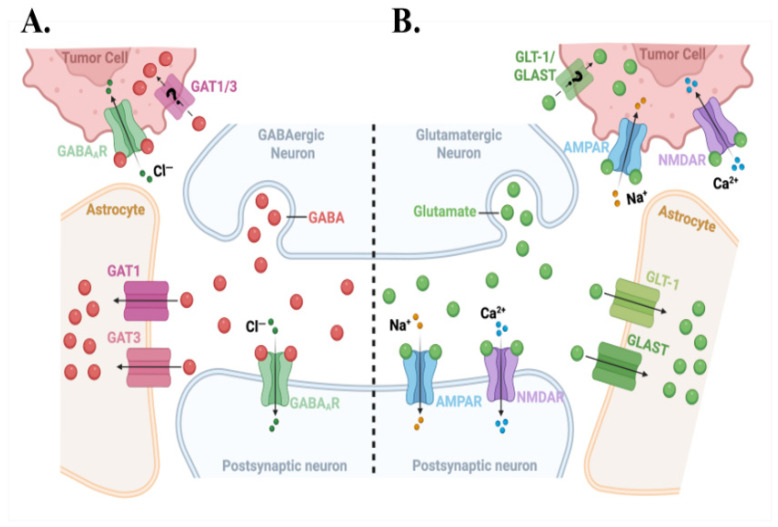
BCBM cells hijack inhibitory and excitatory tripartite synapses. (**A**) Tumor cells abutting GABAergic synapses express functional GABA_A_ receptors and transcripts encoding GAT1 and GAT3 transporters (GAT proteins not shown, yet). This facilitates GABA uptake from presynaptic neurons, and resulting Cl^−^ flux may promote tumor cell quiescence, survival, or migration. GAT1/3 on tumor cells compete with astrocytic GAT1/3 for GABA clearance. (**B**) Tumor cells interfacing with glutamatergic synapses express AMPA and NMDA receptors and likely also GLT-1 and GLAST-like glutamate transporters, allowing them to sense glutamate-dependent Na^+^/Ca^2+^ signaling. By capturing glutamate from the synaptic/peri-synaptic space, tumor cells co-opt neuronal activity-regulated neurotransmission for pro-tumor signaling.

Peri-synaptic positioning places metastatic cells, like brain metastatic breast and melanoma cells, within micrometers of GABAergic (Figure 3A) and glutamatergic synapses (Figure 3B), where they are exposed to and bind neurotransmitters. At glutamatergic synapses, these tumor cells capture glutamate spillover via surface NMDARs (Figure 3B) [16,19]. NMDARs are both ligand-gated (requiring glutamate/D-serine) and voltage-dependent; they remain non-conducting until the postsynaptic membrane is sufficiently depolarized to expel Mg^2+^ (magnesium block) from the channel pore [44,45]. Tumor-associated depolarization may arise from local field effects, AMPAR activation, or voltage-gated ion channels and enable NMDAR opening and downstream Ca^2+^-dependent growth signaling (Figure 3B) [45,46].

**Figure 4:**
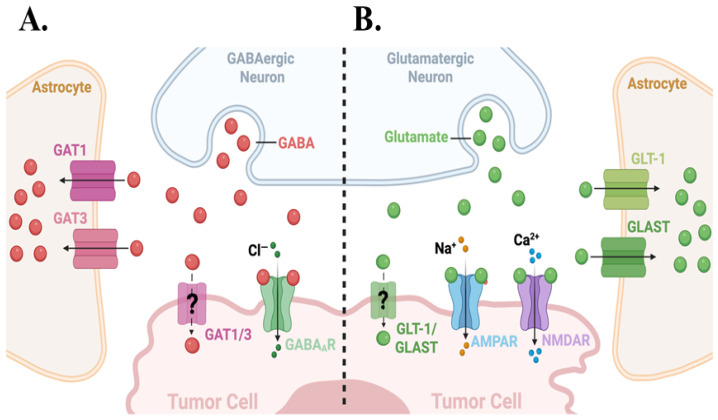
Conceptual model highlighting unresolved mechanisms of tumor integration into inhibitory and excitatory tripartite synapses. (**A**) Tumor cells juxtaposed to GABAergic terminals express GABA_A_ receptors (GABA_A_R) and may also express GAT1/3 transporters. However, the presence, polarity, and functional impact of GAT1/3-mediated GABA transport in tumor cells remain to be confirmed (hence the question mark). (**B**) At glutamatergic contacts (right), tumor cells forming direct synapse-like junctions with neurons harbor AMPA and NMDA receptors, while putative GLT-1 and GLAST-like glutamate transporters (question mark) may facilitate glutamate uptake into tumor cells. Collectively, this leads to Na^+^/Ca^2+^ signaling and metabolic events promoting tumor cell growth and therapy resistance. Our understanding of activity-dependent neuron-tumor coupling is emerging, and many gaps remain to be solved.

Direct neuron–tumor synapses represent a more intimate form of communication. Ultrastructural studies identify synapses with presynaptic vesicles, synaptic clefts, and postsynaptic densities formed directly on tumor cells which contain presynaptic markers (synapsin, synaptophysin, bassoon) and postsynaptic complexes (AMPARs, NMDARs, PSD-95) (Figure 4A,B) [17,18,19,47,48]. Whole-cell patch-clamp recordings in brain-metastatic MDA-MB-231-BR cells reveal spontaneous excitatory currents with kinetics consistent with AMPAR- and NMDAR-mediated synaptic transmission [49].

Neuronal mimicry involves the transcriptional reprogramming of metastatic cells resulting in the upregulation of neuron-specific genes and synaptic components [13,15,18]. Microarray studies show that melanoma cells in the brain upregulate synaptic genes, like *SNAP25*, *SNAP91*, and *Bassoon*, by 100–250-fold compared to those in skin [18]. BCBM express neuroligins (NLGN1, NLGN2), which bind presynaptic neurexins to organize synapses, and re-express SNAP25 which is largely absent from primary breast tumors [13,15,18].

In conclusion, intricate and multilayered strategies enable brain metastatic cells, including BCBM, to establish an architecturally dynamic metastatic niche that encompasses continuous adaptations to progressively exploit the local brain microenvironment.

## 3. GABAergic Metabolic Adaptation

### 3.1. The GABA Shunt as Oncometabolite Pathway

**Figure 5:**
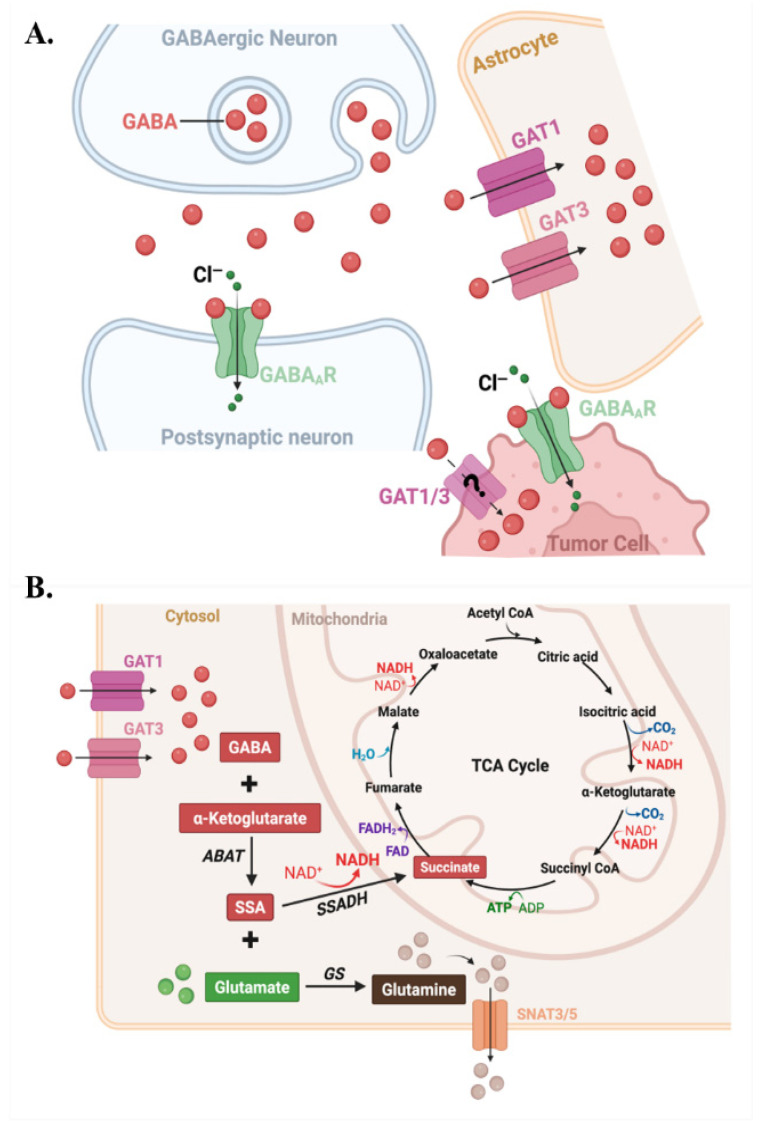
Neuron-astrocyte-tumor GABA shuttling and tumor-intrinsic GABA metabolism. (**A**) GABA released from GABAergic neurons activates postsynaptic GABA_A_ receptors and is cleared by astrocytic GAT1/3. Tumor cells at neuron-tumor contacts express GABA_A_ receptors (GABA_A_R) and GAT1/3 for GABA uptake and Cl^−^ flux. (**B**) Imported GABA enters the GABA shunt, where ABAT and SSADH convert GABA to succinate for the TCA cycle and generate glutamate that can be transformed to glutamine by GS and exported via SNAT3/5.

Human BCBM adopt a GABAergic metabolism by expressing functional GABA_A_ receptors, up-regulating mRNA for GABA transporters GAT1-3 and BGT, and increasing the expression of GABA transaminase (ABAT) [9]. This allows brain metastatic cells to scavenge GABA and convert it into TCA cycle intermediates via the GABA shunt. In this mitochondrial pathway, ABAT enzyme uses α-ketoglutarate as amino acceptor to generate L-glutamate and succinic semialdehyde, which is oxidized by succinic semialdehyde dehydrogenase (SSADH/ALDH5A1) to succinate with concomitant NADH production [9,50,51]. Succinate feeds into the TCA cycle and NADH supports oxidative phosphorylation. When engaged, the GABA shunt provides high-yield energy (Figure 5A,B) [9,50,51].

Clinical BCBM samples show significantly higher ABAT expression than primary breast tumors and normal breast tissue; both HER2+ and TNBC brain metastases display more than two-fold increases in ABAT protein [9]. Access to the synaptic cleft, GABA concentrations can reach millimolar levels during inhibitory neurotransmission, which offers metastatic cells a richer fuel supply than the low micromolar ambient GABA elsewhere in the brain (Figure 3A, Figure 4A and Figure 5A,B) [52,53]. Functionally, exogenous GABA enhances proliferation of TNBC and HER2+ BCBM cells in a concentration-dependent manner. This GABA-mediated growth effect is abolished by the irreversible ABAT inhibitor Vigabatrin, but not by non-metabolizable GABA_A_ agonists such as Muscimol [9]. GABA supplementation increases intracellular NADH by nearly 50%, whereas Muscimol or combined GABA plus Vigabatrin does not, indicating that metabolic catabolism rather than receptor signaling drives this growth advantage [9]. This GABA scavenging strategy is shared by other brain-tropic malignancies. For example, medulloblastoma cells upregulate ABAT and use GABA as an oncometabolite in nutrient-poor cerebrospinal fluid. Non-small cell lung cancer brain metastases activate a FOXA2–ABAT–GABA axis that promotes colonization via NF-κB signaling [11,54].

Collectively, these findings demonstrate that brain metastatic breast cancer cells undergo metabolic reprogramming toward GABA catabolism. This allows these tumor cells to exploit GABA neurotransmitter-rich neural microenvironments and use GABA as an alternative carbon and energy source to fuel proliferation and support the successful colonization of the brain. In addition, brain metastatic BCBM may go one step further by also utilizing GABAergic receptor signaling.

### 3.2. Molecular Components of the GABAergic System

GABA_A_ receptors are pentameric ligand-gated chloride channels assembled from 19 subunit genes. These receptors are frequently upregulated in brain metastases at the transcript and protein level [9]. HER2+ BCBM cells express higher levels of all GABA_A_ receptor mRNA isoforms when compared with non-brain-tropic HER2+ cell lines, and MDA-MB-231-BR TNBC cells upregulate 12 of 15 GABA_A_ subunit transcripts relative to parental cells [9]. Among these subunits, the π subunit GABRP, which can form homo-pentamers, is of particular interest. At physiological GABA levels, GABRP signals via Gα_i/o_ and ERK in a non-tumor context, whereas in cancer cells, GABRP promotes chloride influx and proliferation [55,56]. GABRP is enriched in basal-like tumors that show poor outcomes and have a higher likelihood of brain rather than bone or lung metastasis [56,57,58,59,60]. Silencing GABRP in TNBC cells reduces ERK1/2 phosphorylation, tumorigenicity, migration, and tissue invasion [56]. Additional contributing subunits are GABRA3 (α3), normally CNS-restricted, but aberrantly elevated in HER2+ BCBM, and GABRB3 (β3), an embryonically expressed subunit, co-assembles with GABRA3 to support receptor assembly and surface expression [9,15].

To capture GABA, BCBM cells upregulate multiple GABA transporters [9]. HER2+ brain metastases show elevated transcript levels of the vesicular transporter VGAT (SLC32A1), plasma membrane transporters GAT1-3, and the GABA-betaine transporter BGT. In addition, TNBC brain metastases exhibit at least two-fold increases in VGAT, GAT3, and BGT [9]. GAT1 (SLC6A1), which normally uses the Na^+^/Cl^−^ gradient to clear synaptic GABA in neurons, is expressed at comparable or higher levels on BCBM cells than on surrounding brain cells, enabling direct competition for GABA with neurons [9]. GAT3 (SLC6A11), a classical astrocyte marker, is induced in BCBM cells upon exposure to neurons to further enhance GABA uptake capacity (Figure 4A and Figure 5A,B) [15,61,62].

Together, these data reveal that brain metastatic breast cancer cells co-opt neuronal components of GABA signaling through aberrant expression of GABA_A_ receptor subunits and high-affinity transporters to enhance synaptic GABA uptake. This promotes receptor-driven proliferative signaling and reinforces metabolic and signaling adaptations that enhance BCBM survival and growth within the neuronal niche.

### 3.3. Temporal Dynamics and Therapeutic Implications

Upon seeding in the brain, metastatic BC cells depend on exogenous GABA due to a lack in significant expression of glutamate decarboxylases GAD65 and GAD67 that convert glutamate to GABA [9]. In this initial phase, GABA_A_ receptor activation likely provides survival cues via chloride flux and downstream kinase pathways, such as ERK1/2 and PI3K/AKT [9,55,56]. Concurrently, metastatic cells upregulate ABAT and engage the GABA shunt. This enables them to shift from receptor-mediated signaling to metabolic utilization [9]. Millimolar GABA pulses from interneurons, normally cleared by neuronal GAT1 and astrocytic GAT3, are intercepted by tumor cells expressing these same transporters at comparable or higher levels [9,52,53,62,63].

This temporal shift has therapeutic implications. Valproic acid inhibits enzymes in the GABA degradation pathway, including GABA transaminase/ABAT. This increases intracellular GABA levels and modulates neuronal and potentially tumor cell metabolism [64]. In orthotopic brain tumor xenografts, valproic acid has been shown to prolong survival. However, optimal timing of valproic acid treatment and its specific efficacy in BCBM models remain to be defined [65]. BCBM upregulate multiple GABA pathway components and can use GABA as a metabolic substrate, consistent with neuronal-like adaptation in the brain microenvironment (Figure 5A,B) [9]. Together, these data support a model in which BCBM cells, particularly TNBC, exploit GABAergic signaling and metabolism. There is a clinical need to delineate whether early interventions targeting GABA metabolism in clinically relevant models can improve outcome [9,65].

### 3.4. Breast Cancer Subtype-Specific GABAergic Patterns

GABAergic adaptation is broadly shared among BCBM, but the extent and molecular configurations differ by molecular subtype. TNBC exhibits the most pronounced GABAergic phenotype with especially high GABRP expression and a strong GABA shunt transcriptional signature (ABAT, ALDH5A1, GAT1/SLC6A1, GAT3/SLC6A11) associated with poor survival [15,56]. TNBC brain metastases display approximately three-fold higher GABA_A_ receptor protein levels compared with matched primary tumors and substantial upregulation of most GABA_A_ subunit transcripts, whereas HER2+ BCBM show robust, although somewhat less extreme, GABAergic features [9]. Luminal A and B brain metastases have not been systematically profiled for GABA receptor, transporter, or ABAT expression. It remains unclear whether these BC subtypes rely on alternative neurotransmitter systems or different metabolic strategies for brain colonization. The neuro-metabolic dependencies of luminal A/B brain metastases require further studies to elucidate subtype-specific GABA pathway vulnerabilities that may be exploited for targeted intervention.

## 4. Glutamatergic Signaling and Direct Neuron-Tumor Synapses

### 4.1. Discovery and Structural Characterization

Glutamatergic communication between neurons and metastatic tumor cells displays a spectrum of spatial configurations. This ranges from direct synapses with electron-dense postsynaptic densities [19,66,67], peri-synaptic positioning adjacent to neuron-neuron synapses where tumor cells intercept glutamate in the cleft (Figure 3A,B) [19], and mature pseudo-tripartite arrangements where tumor-associated astrocytes infiltrate these neuron-tumor interfaces and shape extracellular glutamate levels via GLT-1 and GLAST (Figure 4A,B) [68,69,70]. These structural variants likely reflect different stages and strengths of synaptic integration during adaptation of brain metastatic cells to the brain metastatic niche.

**Figure 8:**
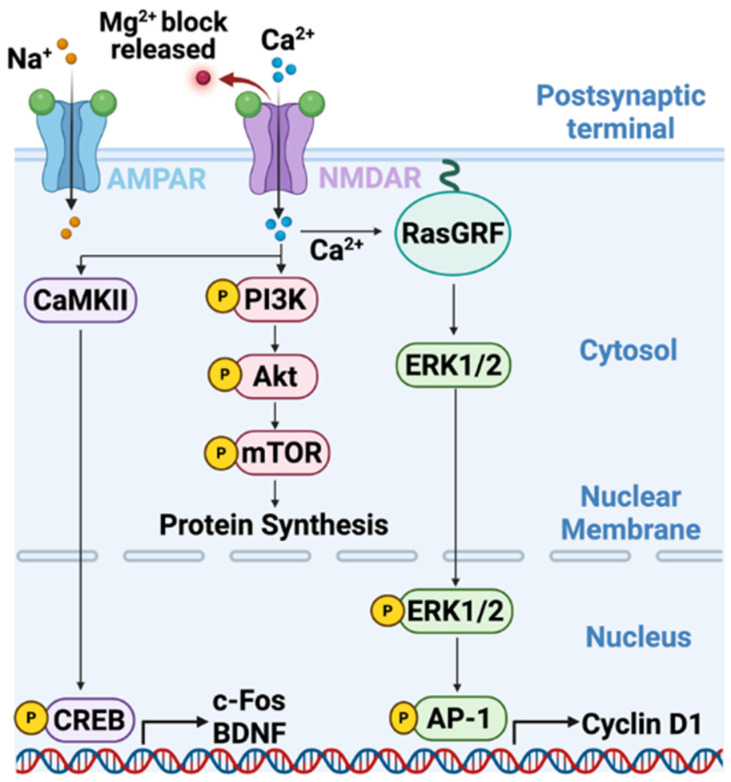
Coordinated AMPAR and NMDAR activation promotes robust postsynaptic Ca2+ influx. Ca2+ entry through NMDARs and Na+ influx through AMPARs engage CaMKII and RasGRF, respectively, driving PI3K-Akt-mTOR and ERK1/2 signaling cascades that converge on nuclear transcription factors (CREB, AP-1) to induce genes such as c-Fos, BDNF, and cyclin D1. This events couple glutamatergic synaptic activity and Ca^2+^ flux to the activation of specific transcriptional programs that result in differential protein synthesis leading to long-term plasticity.

Primary gliomas are well-known for their synaptic connections with cholinergic and glutamatergic neurons to promote glioma cell growth and invasion [47,66,67]. Optogenetic activation of cortical neurons elicits time-locked synaptic responses in nearby glioma cells, consistent with direct functional coupling [66]. Metastatic cancers from breast, lung, and skin also participate in synaptic communication and receive direct input from the brain circuitry [71]. Upon entering the brain, breast cancer and melanoma cells preferentially localize to peri-synaptic niches where they position themselves within micrometers of glutamatergic terminals and express functional NMDARs that capture glutamate spillover (Figure 3B and Figure 5A) [19]. Super-resolution STED imaging visualizes metastatic cells juxtaposed to vGlut2-positive presynaptic terminals that display phosphorylation of GluN2B at Y1472, a hallmark of active NMDAR signaling [16,17]. NMDAR engagement promotes Ca^2+^ influx and activates MAPK/ERK and PI3K/AKT cascades (Figure 8) [19]. Furthermore, electrophysiological recordings indicate that BCBM cells exhibit spontaneous excitatory postsynaptic currents (sEPSCs) with fast kinetics, a characteristic of AMPAR-mediated transmission [16]. Connected via neuronal-tumor cell synapses to the brain circuitry, these metastatic cells may contribute to neurocognitive impairment frequently observed in BCBM patients. This adds circuit dysfunction to the classical mass effects [71,72]. Notably, the pharmacologic blockade with the Alzheimer’s drug and NMDAR antagonist Memantine or GluN2B-selective Ifenprodil, as well as genetic ablation of GluN2B, significantly reduces metastatic burden in mouse models [19]. These findings point out glutamate receptor subunits with specific functions relevant to BCBM.

### 4.2. Glutamate Receptor Composition in BCBM

NMDARs are hetero-tetrameric ion channels composed of two obligatory GluN1 subunits and two modulatory GluN2A-D or GluN3A-B subunits [73,74]. GluN1 is ubiquitously expressed in neurons and carries the glycine-binding site. Expression of GluN1 is also detected in BCBM [73,74,75]. Particularly in TNBC brain metastases, GluN2B (encoded by the *GRIN2B* gene) is preferentially elevated, and high *GRIN2B* gene transcription correlates with worse distant relapse-free survival [19]. Immunohistochemistry reveals that a large fraction of patient-derived brain metastases exhibit membrane staining for phosphorylated GluN2B (Y1472 and Y1252) and associated postsynaptic components. Intriguingly, pGluN2B positivity in BCBM far exceeded that of matched primary tumors [19,69]. Functionally, GluN2B-containing NMDARs display slower deactivation kinetics than GluN2A-containing receptors, suggesting potentially prolonged receptor signaling [76,77]. Like their neuronal counterparts, NMDARs in the membrane of BCBM are strongly voltage-dependent and require membrane depolarization to allow Ca^2+^ permeation [78,79].

AMPARs, composed of GluA1-4 subunits, mediate fast excitatory currents and are expressed in both glioma and brain metastases [17,80]. In BCBM and melanoma brain metastases, GluA1 and GluA2 subunits are prominent [17]. The presence or absence of GluA2 critically influences Ca^2+^ permeability. GluA2-lacking AMPARs are Ca^2+^-permeable, whereas GluA2-containing receptors largely exclude Ca^2+^ but remain highly conductive to Na^+^ and K^+^ [81,82]. Patch-clamp recordings from brain-metastatic cells show fast EPSCs with rise times < 1 ms and decay constants of approximately 5–10 ms which is characteristic of AMPAR-mediated transmission sensitive to pharmacological block by NBQX and Perampanel [17,83].

Many key questions remain regarding the dynamic interplay between AMPAR and NMDAR signaling during the evolution of brain metastases. It is unknown whether the receptor subunit composition shifts as micro-metastatic clusters evolve into macroscopic tumors. Furthermore, how these receptor subunit shifts affect synaptic sensitivity and therapeutic responsiveness remains elusive.

### 4.3. Breast Cancer Subtype-Specific Glutamatergic Receptor Composition

TNBC brain metastases exhibit robust glutamatergic receptor expression, with particular reliance on GluN2B [19]. Primary TNBC tumors with brain tropism show higher *GRIN2B* gene expression than primary TNBC tumors that metastasize to bone or lung, suggesting *GRIN2B* transcriptional regulation is part of a brain homing and/or outgrowth expression signature [19]. When co-cultured with cortical neurons, the enhanced proliferation of brain-metastatic MDA-MB-231-BR cells is abolished by treating TNBC cells with NMDAR antagonists or GluN2B knockdown, indicating functional dependency of these TNBC cells on neuronal glutamate signaling [19]. TNBC brain metastases also express AMPAR subunits, including GluA2, and may harbor Ca^2+^-permeable AMPARs, but their relative contributions to growth versus migration remain to be defined. HER2+ BCBM show moderate glutamatergic receptor expression and GluN2B levels are generally lower than in TNBC [19]. It is currently unknown whether NMDAR signaling synergizes with canonical HER2 pathways or is partly redundant. For luminal subtypes, available data is limited. Luminal A and B tumors have lower incidence and longer latency to CNS relapse compared with TNBC and HER2+ disease. Although luminal brain metastases can express glutamate receptors, expression levels are lower than in basal-like breast cancers, and their functional roles remain largely unexplored. TCGA analyses suggest a modest association between *GRIN2B* expression and metastasis-free survival in Luminal A tumors [19].

## 5. Synaptic Adhesion Molecules and Tumor–Astrocyte Coupling

In the healthy brain, synaptic adhesion molecules coordinate the assembly and specialization of excitatory and inhibitory synapses [84]. Neuroligins (NLGNs) on the postsynaptic membrane bind presynaptic neurexins and contribute to the synaptic cleft matrix and specify synapse identity. NLGN1 and NLGN2 preferentially associate with excitatory glutamatergic synapses and inhibitory GABAergic synapses, respectively [85,86]. In BCBM, data on synaptic adhesion molecules are limited but suggest similar molecular functions at the BCBM–neuron interface. MDA-MB-231-BR brain metastases display ultrastructural pseudo-synaptic contacts with glutamatergic neurons, characterized by asymmetric excitatory-type synapses with vesicle-rich presynaptic boutons and electron-dense postsynaptic densities [19]. Core postsynaptic scaffolding proteins detected in these metastatic lesions include PSD-95 and adhesion molecules, such as NLGN2, and shows the remarkable ability of BCBM cells for neuronal mimicry by assembling neuron-like postsynaptic specializations [19].

Neural cell adhesion molecule (NCAM) is a key regulator of neural migration and plasticity [87]. NCAM function is modulated by polysialic acid (PSA) modification, and PSA-NCAM carries a large negative charge that reduces cell–cell adhesion and promotes motility [88]. In the brain, PSA-NCAM is normally restricted to developmental and plastic regions such as the hippocampus [89,90]. In glioblastoma and pediatric brain tumors, high PSA-NCAM expression correlates with aggressive tissue invasion [91,92,93]. In BCBM, the re-expression of PSA-NCAM is also linked to invasive behavior, but data across subtypes and clinical cohorts require further confirmation [94,95].

Protocadherin-7 (PCDH7) emerges as a critical mediator of physical coupling between brain metastatic cells and astrocytes via connexin-43 (Cx43) gap junctions [96]. These Cx43 hemichannels form at tumor–astrocyte interfaces and enable bidirectional exchange of small signaling molecules, including cGAMP [96]. Transfer of TNBC tumor-derived cGAMP into astrocytes activates the STING pathway and prompts reactive astrocytes to secrete interferons (IFNα, IFNβ) and TNF. These cytokines activate proinflammatory STAT1 and NF-κB signaling in TNBC cells contributing to their enhanced survival and chemoresistance [96]. Pharmacologic disruption of these gap-junctional contacts with agents such as Meclofenamate or Tonabersat significantly reduces brain metastatic burden in preclinical models and highlights synaptic and gap-junction adhesion complexes as actionable vulnerabilities [96].

## 6. Astrocyte Reprogramming and the Pseudo-Tripartite Niche

Astrocytes are central to maintaining neurochemical stability by regulating inhibitory and excitatory neurotransmission [97,98]. Astrocytic GABA transporters GAT1/SLC6A1 and GAT3/SLC6A11 control ambient GABA levels and inhibitory tone (Figure 4A) [98]. Astrocytes also express high-affinity glutamate transporters GLAST/EAAT1 and GLT-1/EAAT2 to keep extracellular glutamate at low resting concentrations (~1 μM) to prevent excitotoxicity (Figure 4B) [97].

**Figure 6:**
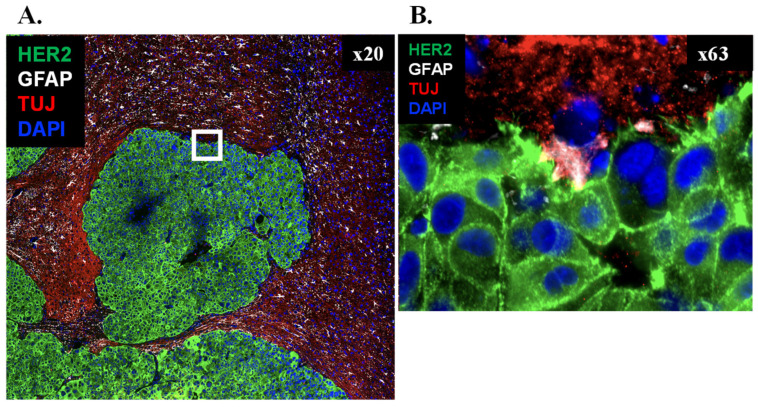
HER2+ brain metastasis at the tumor-brain interface. (**A**) Overview (×20) of a HER2+ metastatic nodule (green) surrounded by GFAP+ reactive astrocytes (white) and TUJ (βIII-tubulin, TUBB3)-positive neurite-like processes (red) within the adjacent brain parenchyma. TUJ is used here as a neurite-enriched marker, acknowledging that βIII-tubulin/TUBB3 can be induced in non-neuronal cells under pathological conditions. (**B**) Higher magnification (×63) of the boxed region in A shows close apposition of HER2+ BCBM cells (green) with interdigitating GFAP+ astrocytic processes (white) and adjacent TUJ-positive neurite-like profiles (red). DAPI (blue) served as nuclear stain.

In the metastatic setting, astrocytes undergo profound transcriptional and functional reprogramming. In the presence of brain tumor cells, astrocytes adopt reactive, immune-modulating, pro-metastatic, and spatially specialized states, reflecting a transition from homeostatic protectors to active collaborators (Figure 6) [99,100,101,102]. Early in brain invasion, GFAP^+^/vimentin^+^ reactive astrocytes form cellular barriers around nascent tumor clusters and secrete anti-tumoral factors, such as plasminogen and FasL, to restrict outgrowth [103]. Successful colonization depends on the ability of metastatic cells to subvert these astrocytic defenses and reprogram astrocytes into pro-tumoral “partners in crime” [96,103].

Once reprogrammed, astrocytes employ an arsenal of multiple strategies to promote tumor survival and expansion. Intravital imaging shows astrocytic processes infiltrating deeply into tumor margins, underscoring structural integration [99,103,104]. Pro-metastatic astrocytes secrete growth-promoting cytokines such as CCL2, IL-6, and IL-23 and connect to tumor cells via Cx43 gap junctions and protocadherin-7 mediated contacts [96,105,106,107]. Elevated astrocytic lactate-pyruvate shuttling and glutamate-glutamine shunt contribute to enhanced tumor metabolic fitness [70,108,109]. In parallel, astrocytic serpins (SERPINA1, SERPINB2) prevent plasminogen-dependent cell death and facilitate vascular co-option to allow tumor cells to exploit existing vasculature [103,110].

Astrocytes also participate in the pseudo-tripartite synaptic niche at neuron-tumor interfaces. Under physiological conditions, neuronal glutamate is taken up by astrocytes and converted by glutamine synthase to glutamine. Glutamine is released from astrocytes primarily via glutamine exporters, the primary being the system N transporters SNAT3 (SLC38A3) and SNAT5 (SLC38A5), with additional contribution from Na^+^-independent system L transporters such as LAT1 under certain metabolic conditions [111,112,113] (Figure 5B) [114]. Brain metastatic cells can upregulate glutamine transporters and utilize glutamine as an oncometabolite to support their anabolic metabolism and growth [115]. At some sites, tumor cells usurp positions normally occupied by peri-synaptic astrocytic processes at glutamatergic synapses and express NMDARs to exploit glutamate released from pre-synaptic terminals as a trophic signal (Figure 3B) [19]. This displacement and repurposing of astrocytic functions not only deepens the structural and metabolic integration of tumor cells into neural circuits but preserves and/or enhances synaptic glutamate supply required for NMDAR-mediated growth signaling.

Single-cell and spatial profiling have revealed multiple astrocyte subpopulations in tumor-bearing brain tissue, but their specific roles in tumor-astrocyte-neuron interactions are currently incompletely understood [100,101,102]. High-resolution temporal and spatial mapping of astrocyte phenotypes in BCBM, including subtype-specific patterns across TNBC, HER2+, and luminal metastases, is clearly needed to delineate astrocytic states most critical for supporting tumor growth and therapeutic resistance [116,117]. Similarly, the functional impact of targeting neurotransmitter transporters and metabolic shuttles at brain metastatic niches and the potential of drugs that modulate neurotransmission and astrocyte activation remain largely unexplored and represent important areas for future work.

## 7. Calcium Is a Signaling Messenger in Brain Metastatic Cells

### 7.1. From Synaptic Excitability to Proliferation

Calcium is a universal intracellular messenger that orchestrates neuronal excitability, synaptic plasticity, and memory formation [118]. At the brain metastatic niche, breast cancer cells use NMDAR mediated Ca^2+^ influx to drive proliferation, invasion, and therapy resistance [19,67,119]. Hence, physiological glutamatergic Ca^2+^ signaling normally supporting neuronal function becomes an oncogenic driver in BCBM.

In normal breast epithelium, plasma membrane Ca^2+^ channels are sparsely expressed and powerful calcium pumps maintain resting cytosolic Ca^2+^ near 100 nM [120]. Malignant transformation commonly coincides with the upregulation of Ca^2+^ influx channels and altered clearance mechanisms to reshape the intracellular Ca^2+^ landscape [120,121]. Brain metastatic cells expressing functional NMDARs at peri-synaptic or synaptic contacts with neurons utilize glutamate-driven NMDAR activation to enable millisecond bursts of Ca^2+^ influx, which trigger seconds of activation of Ca^2+^-dependent kinases, such as CaMKII and PKC Ca^2+^ signaling networks [19,46]. This results in the activation of transcriptional programs to promote pro-growth and pro-survival phenotypes in metastatic cells [118,120,121,122].

### 7.2. Cooperative AMPAR-NMDAR Regulation of Calcium Influx

NMDAR channel opening is a multi-step process that involves binding glutamate and a co-agonist (glycine or D-serine) to the ubiquitous NMDAR subunit GluN1. Even with both sites occupied, the pore remains blocked by extracellular Mg^2+^ at resting potentials (approximately −65 to −70 mV) [73,74,78,79] as membrane depolarization is required to dislodge Mg^2+^ to permit Ca^2+^, Na^+^, and K^+^ flux [78,79]. This voltage dependence means that metastatic cells are unlikely to experience significant NMDAR-mediated Ca^2+^ influx until they also engage depolarizing membrane conductance.

**Figure 7:**
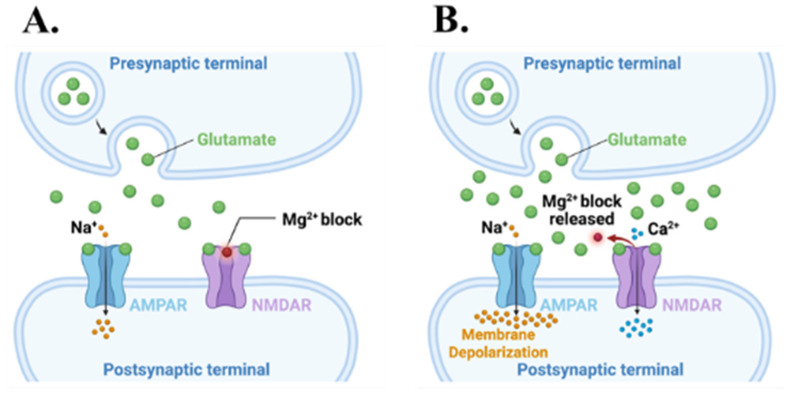
AMPAR-NMDAR co-activation and downstream signaling. (**A**) Glutamate binding to AMPAR alone permits Na+ influx but leaves NMDAR channels blocked by Mg^2+^. (**B**) Sufficient AMPAR-mediated depolarization relieves the NMDAR Mg^2+^ block and allows Ca^2+^ entry upon glutamate binding.

A working model extrapolated from neuronal LTP posits that AMPAR activation is a way to promote the necessary membrane depolarization [33,118,123,124]. While metastatic cells early during brain colonization may seek closer proximity to active glutamatergic synapses and encounter spillover glutamate, their membrane potential remains near −70 mV, leaving NMDARs largely Mg^2+^-blocked [19,46]. Progressive AMPAR expression could allow AMPAR-mediated Na^+^ influx, thereby shifting the membrane potential toward −40 to −30 mV, relieving the Mg^2+^ block and enabling robust Ca^2+^ entry through NMDARs [118,123,124]. This cooperative AMPAR-NMDAR mechanism is well established in neurons (Figure 7A,B); whether metastatic cells fully recapitulate it and on what timescales remain critical open questions of clinical relevance that need to be answered.

### 7.3. Calcium Microdomains and Downstream Signaling Cascades

Within 10–100 nm of an open NMDAR channel pore, a jet of Ca^2+^ influx creates transient, highly localized microdomains in which intracellular Ca^2+^ concentrations can rise from ~100 nM to low micromolar levels [125,126]. This selectively activates Ca^2+^-sensitive enzymes, such as CaMKII, calcineurin, and Ca^2+^-activated adenylyl cyclases, that are tethered nearby these microdomains [125,127]. CaMKII, a central decoder of NMDAR-mediated Ca^2+^ signals, auto-phosphorylates at Thr286 within these microdomains and remains active after Ca^2+^ returns to baseline. These kinetics transform the transient pulsatile synaptic activity into regulated activation of signaling pathways [36,127,128]. This transformation begins with activated CaMKII phosphorylating the AMPAR subunit GluA1 at Ser831. This increases single-channel conductance and promotes further AMPAR membrane insertion [37,38]. A Ca^2+^ driven positive feedback loop is established in which increased AMPAR membrane content enhances depolarization to amplify NMDAR-mediated Ca^2+^ influx and elevated CaMKII activation (Figure 7A,B) [37,38,118], reminiscent of synaptic LTP in neurons. In BCBM, analogous mechanisms may strengthen neuron-tumor synaptic connections and intensify growth-promoting signals in BCBM [19]. Next, CaMKII couples NMDAR-mediated Ca^2+^ influx to ERK1/2 activation through Ca^2+^-dependent Ras guanine nucleotide exchange factors, such as RasGRF1/2, to promote cyclin D1 expression and G1 to S progression (Figure 8) [129,130]. Ca^2+^-activated adenylyl cyclases (AC1 and AC8) generate cAMP, which activates PKA and CREB phosphorylation at Ser133 [131,132,133]. CREB^S133^ drives transcription of immediate-early genes such as c-Fos, Arc, brain-derived neurotrophic factor (BDNF), and genes encoding synaptic proteins SNAP25, neuroligins, and synaptophysin (Figure 8) [131,132,133,134,135]. This transcriptional program promotes neuronal mimicry by consolidating the capacity of metastatic cells to form and maintain synaptic contacts.

The Ca^2+^/calmodulin-dependent phosphatase calcineurin opposes CaMKII kinase activity by promoting AMPAR dephosphorylation and receptor endocytosis, which favors synaptic depression [41,136]. The relative enzymatic activities of CaMKII and calcineurin determine whether synaptic inputs strengthen or weaken synaptic connectivity [41,118]. Perturbations of this balance in metastatic cells, e.g., by sustained CaMKII activation and/or reduced calcineurin activity, can likely bias neuron-tumor synaptic communication toward a persistent potentiation and continuous BCBM growth signaling [19,118]. This is an important area for future studies as information is currently lacking on the CaMKII/calcineurin balance in TNBC, HER2+, and luminal BCBM [19,46].

## 8. AMPARs and Voltage-Gated Calcium Channels Amplify and Diversify Intracellular Ca^2+^ Signaling

### 8.1. AMPARs as Depolarization Initiators

AMPAR subunits GluA1-4 are expressed in BCBM and contribute to fast excitatory currents [66,67,80]. AMPAR opening produces rapid Na^+^ influx and K^+^ efflux which unlocks NMDARs and activates voltage-gated calcium channels (VGCCs) [46,118,137]. As the membrane potential in neurons shifts from approximately −70 mV toward −40 mV, L-type (Cav1.2, Cav1.3) and T-type (Cav3.x) VGCCs open and further augment Ca^2+^ entry [46,137]. Compared with their primary BC tumor counterparts, BCBM upregulate VGCCs, enabling them to translate brief synaptic depolarizations into sustained Ca^2+^ elevations [46].

The degree to which AMPAR and/or VGCC facilitation of Ca^2+^ influx contributes to the proliferation of brain metastases remains uncertain [66,67]. Data from neuronal systems suggest that Ca^2+^-permeable (GluA2-lacking) AMPARs may preferentially regulate local cytoskeletal dynamics and migration, whereas NMDAR-derived Ca^2+^ influx more strongly influences nuclear transcriptional programs [19,118,125]. Whether a similar division of functionalities exists in BCBM has not been tested directly.

### 8.2. VGCCs and Mitochondrial Coupling

Ca^2+^ influx via VGCCs outlasts ligand-gated receptors and tends to integrate stimuli over hundreds of milliseconds [46,137]. Ca^2+^ signaling is closely linked to mitochondrial activity. Mitochondria cluster near VGCCs and NMDARs and rapidly take up Ca^2+^ via the mitochondrial Ca^2+^ uniporter [138]. Mitochondrial Ca^2+^ stimulates key TCA cycle enzymes, such as isocitrate dehydrogenase and α-ketoglutarate dehydrogenase, resulting in increased NADH production and ATP synthesis [138]. In brain metastases, sustained Ca^2+^ influx via NMDARs and VGCCs likely elevates mitochondrial ATP output chronically to meet the high energetic demands of continuous proliferation and adaptation [46,137,138].

A coherent picture is starting to emerge where AMPAR-driven membrane depolarization opens VGCCs to enhance mitochondrial metabolism, while NMDAR microdomain Ca^2+^ signals activate kinase and transcriptional cascades [19,66,67,137]. By decoding the different Ca^2+^ signals and integrating their distinct Ca^2+^ sources, metastatic cells magnify neuronal input into intricate and robust growth and survival programs.

### 8.3. Calcium Influx Impacts Proliferation, Survival, and Therapy Resistance

In TNBC brain metastases, NMDAR activation drives ERK1/2 and PI3K/AKT signaling and upregulates cyclin D1 to promote G1 to S cell cycle transition and proliferation (Figure 8) [19,46], with GluN2B subunit found to be critical for brain metastatic burden in mouse models [19]. Co-culture of TNBC cells with cortical neurons enhances TNBC proliferation. This effect is abrogated by NMDAR antagonists which supports the functional dependency on neuronal glutamate [19,46].

Ca^2+^ influx also strengthens survival signaling. Activation of PI3K/AKT/mTOR by Ca^2+^ promotes the phosphorylation of pro-apoptotic BAD and the upregulation of anti-apoptotic Bcl-2 family members, resulting in resistance to cell death [139,140]. In glioma, glutamatergic signaling reinforces DNA damage repair pathways and anti-apoptotic programs to foster resistance against radiotherapy and chemotherapy [104,141]. Whether BCBM leverage similar Ca^2+^-dependent survival mechanisms has not been comprehensively studied but is a plausible extension that merits investigation [19].

## 9. Emerging Frontiers and Knowledge Gaps

### 9.1. Intercellular Communication Platforms Beyond Synapses and Gap Junctions

**Figure 9:**
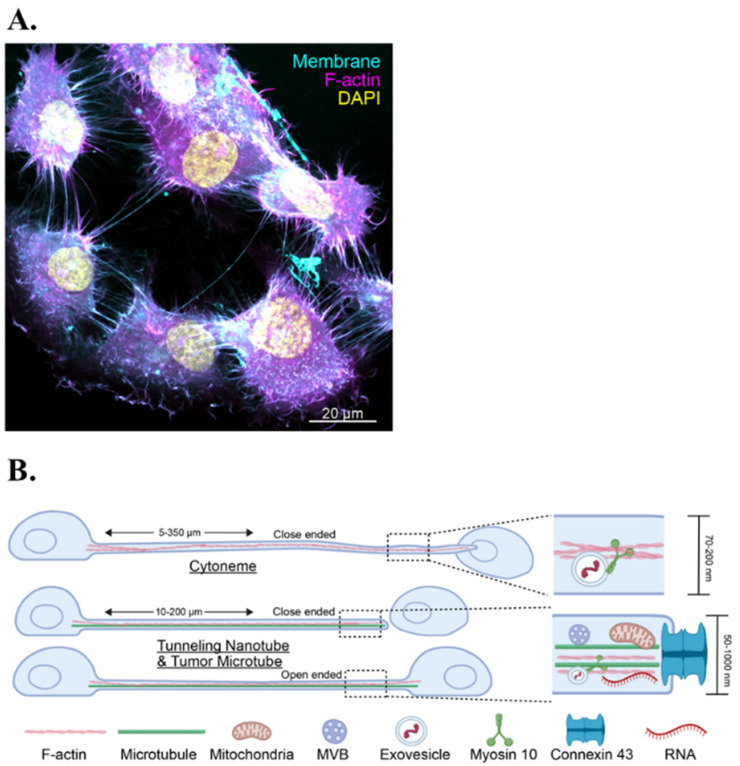
Tumor cell membrane protrusions and long-range intercellular conduits. (**A**) Fluorescence microscopy of cultured BCBM cells showing F-actin rich membrane protrusions connecting neighboring BCBM cells. (**B**) Schematic representation distinguishing thin, close-ended cytonemes facilitating morphogen transport from thicker tunneling nanotubes/tumor microtubes. The latter can be close- or open-ended and support long-distance exchange of organelles, vesicles, and signaling molecules between tumor cells.

Brain tumors deploy specialized membrane extensions, named cytonemes, tunneling nanotubes (TNTs) and tumor microtubes (TMs) enabling them to form an interconnected network among tumor cells and between tumor cells and resident cells in the brain microenvironment (Figure 9A,B) [142,143]. Cytonemes are a class of long, filopodia-like protrusions that specialize in the transport and reception of morphogens and growth factors (Figure 9B) [144,145]. Emerging work suggests cytonemes may contribute to communications in primary and metastatic brain tumors, including BCBM, by directional transport of growth factor signals that remodel the brain microenvironment (Figure 9A) [146,147,148,149]. Because cytonemes are thin and transient, visualizing these structures requires advanced sectional imaging.

Tunneling nanotubes (TNTs) and tumor microtubes (TMs) are long, thin, actin- or microtubule-based conduits that create multicellular networks across tumors. This allows direct cytoplasmic transfer of organelles, signaling proteins, RNAs, and ions from one cell to the next (Figure 9B) [143,150]. In glioblastoma, TNT/TM networks support adaptation by propagating Ca^2+^ waves, sharing mitochondria, and redistributing metabolic resources, all of which enhance survival and resistance to therapy [104,143,151]. Recently, the neuron-to-cancer cell transfer of mitochondria was shown for 4T1 mouse TNBC cells. 4T1 brain metastases showed a significantly higher percentage of breast cancer cells with neuron-donated mitochondria, suggesting a metabolic advantage in the brain [152].

The precise roles of cytonemes and TNT/TM in BCBM remain speculative and are currently under investigation [146,153]. Targeting these cytoneme and TNT/TM networks may disrupt long-range communication within metastatic lesions and between tumor and stromal cells, thus, sensitizing BCBM to chemo- and radiotherapy [104,143,151]. However, specific druggable targets and the therapeutic index of such interventions remain undefined.

### 9.2. Unanswered Questions in Neuron–Tumor Calcium Signaling

Most mechanistic insights into neuron-tumor Ca^2+^ signaling in BCBM derive from a single TNBC model, MDA-MB-231-BR, Notably, subtype-specific differences are largely unexplored [19,46]. Constitutively elevated HER2 kinase activity in HER2+ BCBM and estrogen receptor signaling in luminal BCBM may use alternative Ca^2+^ entry routes, such as store-operated Ca^2+^ entry (SOCE) [154,155]. Systematic comparisons of NMDAR, AMPAR, VGCC, and SOCE contributions across TNBC, HER2+, and luminal brain metastases are lacking.

Another open question is the role of Ca^2+^-permeable (GluA2-lacking) AMPARs in early micro-metastases in the brain [19,66,67]. These receptors may facilitate an initial depolarization and Ca^2+^ influx before robust NMDAR expression is established in macro-metastases. It remains unknown whether these GluA2-lacking AMPARs are sufficient to drive proliferation or mainly serve as facilitators for later NMDAR-dependent growth [19,66]. Real-time in-vivo visualization of Ca^2+^ microdomains and Ca^2+^ flux imaging in BCBM cells in response to neuronal activity is also missing. Employing two-photon microscopy combined with genetically encoded Ca^2+^ indicators would elucidate the important temporal relationship between neuronal firing, Ca^2+^ flux, and behavioral states [19,125,126].

Finally, how CaMKII/calcineurin balance varies spatially and temporally within metastatic lesions is unknown [41,118,136]. Heterogeneous CaMKII/calcineurin ratios could generate microdomains of high and low synaptic dependence within the same lesion, which may generate spatially distinct resistance profiles to NMDAR-targeted therapies.

### 9.3. Clinical Translation

NMDAR antagonists Memantine (already approved for neurodegenerative diseases) and Ifenprodil show efficacy in preclinical models of brain metastasis, particularly in *GRIN2B*-high TNBC [19]. Clinical translation is hampered by the lack of biomarkers to identify NMDAR-dependent lesions and by concerns regarding CNS toxicity at doses required for anti-tumor effects [19,156]. Advanced imaging modalities, including glutamate-weighted MRI (GluCEST) and PET tracers for NMDAR density (e.g., ^18^F-GE-179), may help stratify patients and confirm drug-receptor engagement in-vivo [157,158]. Cerebrospinal fluid (CSF) profiling for glutamate, Ca^2+^-related proteins, or astrocytic markers (e.g., S100B) might complement imaging as minimally invasive biomarkers of neuronal-tumor activity and treatment response [159]. Integrating such biomarkers into future trials will be essential to match patients with appropriate NMDAR- or glutamatergic-targeted therapies and to refine dosing paradigms that achieve sufficient brain exposure with acceptable side-effect profiles [19,156,159].

In breast cancer brain metastases (BCBM), the only systemic agents with robust, trial-level clinical evidence are HER2-targeted therapies in HER2-positive disease, rather than neuron-directed repurposed drugs. In the phase II HER2CLIMB trial (NCT02614794) [160], adding tucatinib to trastuzumab and capecitabine significantly improved overall survival and intracranial progression-free survival versus trastuzumab–capecitabine alone in patients with HER2-positive metastatic breast cancer, nearly half of whom had brain metastases, including active lesions, and led to regulatory approval of this triplet for patients with or without brain metastases. Antibody-drug conjugates such as trastuzumab deruxtecan have also shown meaningful intracranial activity in HER2-positive advanced breast cancer with stable or active brain metastases in phase II/III studies and a large phase 3b/4 trial (e.g., DESTINY-Breast program) [161,162].

Repurposing of drugs originally developed to treat non-tumor brain neurological conditions for the treatment of tumors in the brain is an expedient, economical, and valid clinical option, as exemplified for antiepileptic drugs [163]. Clinically available neuroactive agents that are mechanistically attractive for targeting neuron–tumor circuits, such as the NMDA antagonist memantine or the AMPA antagonist perampanel, are currently used only as supportive therapies. Memantine was used in RTOG-type WBRT trial NRG-CC001 (NCT02360215) [164] (Table 1). No clinical trial has yet demonstrated an antitumor effect of AMPA/NMDA blockade or Trk inhibition specifically in BCBM, beyond case-level and small-cohort reports of intracranial responses with TRK inhibitors, such as larotrectinib and entrectinib in rare NTRK-fusion positive solid tumors with brain metastases [165,166] (Table 1). Repurposed macitentan targeting endothelin receptors to disrupt astrocyte signaling at the brain metastatic niche remains at an experimental stage [167,168] (Table 1). There is a clear gap between strong preclinical data on neuronal mimicry and neuron-tumor synapses. The present clinical trial portfolio in BCBM remains dominated by HER2-directed systemic strategies such as tucatinib-based triplets (HER2CLIMB, NCT02614794) [160] and trastuzumab deruxtecan (DESTINY-Breast program) [161,162].

### 9.4. Current Knowledge Gaps

The diverse spectrum of HER2+ BCBM is much less investigated for their neurotransmitter dependencies compared to TNBC. There is an urgent need for future experimental interrogations into neurotransmitter-elicited metabolic and cellular signaling mechanisms in HER2-enrichend and luminal B-derived BCBM. While luminal A BC are less likely to develop BCBM (6–7%) than TNBC and HER2+ BC [169,170], luminal A molecular subtype is most frequent (50–60%) [171], accounting for a significant number of BCBM patients. There is a lack of experimental luminal A BCBM models severely limiting fundamental and translational research efforts.

The presence and functional impact of GABA and glutamate transporters in BCBM tumor cells is still not fully understood. In part, this is based on the scarcity of suitable in-vivo methods for the detection and functional assessment of tumor cell transporters in xenograft models of BCBM. BCBM cells grown in-vitro do not fully recapitulate in-vivo receptor expression and neurotransmitter responses. Our current lack of understanding possible roles of HER2 signaling pathways in affecting neuronal mimicry phenotypes, the regulation and/or receptor crosstalk with GABAergic/glutamatergic receptors, the regulation of calcium signaling pathways promoting growth and therapy resistance, or sustained long-distance cell-cell communication in the brain metastatic niche highlights an urgent need for future exploration of this clinically highly relevant topic at this neuroscience—neuro-oncology frontier.

## 10. Concluding Remarks

Breast-to-brain metastases co-opt core principles of neurobiology, including neurotransmitter signaling, synaptic plasticity, and astrocyte-mediated homeostasis, to construct a metastatic niche that is both structurally integrated and metabolically optimized. Early BCBM colonizers rely heavily on GABA as an oncometabolite (Figure 5A,B) and on modest peri-synaptic glutamatergic cues (Figure 3B). As brain metastatic lesions mature, the tumor cells exploit direct glutamatergic synapses, NMDAR/AMPAR signaling, VGCC-mediated Ca^2+^ influx, and mitochondrial coupling to sustain proliferation and resist therapies (Figure 4B and Figure 8) [9,19,46,137,138]. Reprogrammed astrocytes, gap junction-mediated cGAMP-STING signaling, synaptic adhesion molecules, and long-range intercellular extensions (TNTs, TMs, cytonemes) further embed (breast cancer) metastatic cells within the brain circuitry (Figure 6A,B and Figure 9A,B) [103,142,143,146]. Future research is urgently needed to better understand long cellular connections between cancer cells and resident brain cells that may facilitate targeted placements of signaling molecules and organelles or membrane receptors to facilitate selective communication between tumor cells and surrounding brain cells.

In conclusion, the breast cancer subpopulations that establish brain metastases do so through a dynamic process of adaptation, progressive integration, and insidious reprogramming of resident brain cells to create a unique local brain metastatic niche microenvironment.

## Figures and Tables

**Table 1:** Brain metastasis–focused neuron–tumor circuit drugs (including BCBM).

Drug	Status in Brain Mets (Clinical vs. Experimental)	Primary Target	Role in Neuron–Tumor Interactions	Brain Met/BCBM Clinical Context (Trial IDs Where Applicable)	Key DOI(s)
Memantine	Clinical trials/use in brain metastases	NMDA receptor antagonist	May attenuate NMDAR-dependent neuron–tumor signaling; clinically used as neuroprotection, not antitumor therapy.	Tested in WBRT ± hippocampal-avoidance trials that include brain met patients, e.g., NRG-CC001 (NCT02360215).	[164]
Perampanel	Antitumor evidence remains preclinical; clinical use (supportive) in brain tumors/metastases; no antitumor trial yet	AMPA receptor antagonist	Blocks AMPA-mediated excitatory synapses; preclinical work shows reduced brain metastatic burden in breast/melanoma models.	Widely used for seizure control in patients with primary and metastatic brain tumors; no registered trial testing antimetastatic efficacy in BCBM.	[16]
Larotrectinib	Clinical trials and routine use in NTRK-fusion tumors with brain mets	TrkA/B/C inhibitor	Interferes with neurotrophin-driven neuron–tumor crosstalk; shows CNS penetration and intracranial responses.	Basket trials in NTRK-fusion–positive solid tumors; intracranial responses in patients with brain mets, including rare breast cancer cases.	[165]
Entrectinib	Clinical trials and use in fusion-positive tumors with brain mets	TrkA/B/C, ROS1, ALK inhibitor	Similar rationale to Larotrectinib, with robust CNS activity in fusion-positive cancers.	Phase I/II basket trials with brain-met cohorts (e.g., ROS1/NTRK-positive NSCLC and other tumors); breast-cancer cases are uncommon but eligible.	[166]
Macitentan	Limited clinical exploration; mainly preclinical for brain mets	Endothelin receptors (ET_A/ET_B)	Targets astrocyte–tumor protective signaling in the brain microenvironment.	Repurposing suggested for brain mets (including BCBM) based on preclinical data; no established BCBM-specific clinical trial.	[167,168]

## Data Availability

No new data were created or analyzed in this study.
